# Development and Implementation of Liposomal Encapsulated Micronutrient Fortified Body Oil Intervention for Infant Massage: An Innovative Concept to Prevent Micronutrient Deficiencies in Children

**DOI:** 10.3389/fpubh.2020.567689

**Published:** 2021-01-25

**Authors:** Aditi Apte, Himangi Lubree, Mudra Kapoor, Sanjay Juvekar, Rinti Banerjee, Ashish Bavdekar

**Affiliations:** ^1^Vadu Rural Health Program, King Edward Memorial Hospital Research Centre, Pune, India; ^2^Nanomedicine Laboratory, Department of Biosciences and Bioengineering, Indian Institute of Technology Bombay, Mumbai, India

**Keywords:** causal pathway, theory of change, implementation science, translational, scale–up

## Abstract

Indian communities have the ancient cultural practice of gentle oil massage for infants which has been shown to play a beneficial role in neuro-motor development. The concept of incorporating nanosized liposomes of micronutrients (i.e., iron, folate, vitamin B12, and vitamin D) in the body oil leverages this practice for transdermal supplementation of essential micro-nutrients. This paper describes the experience of developing an intervention in the form of body oil containing nanosized liposomes of iron and micro-nutrients built on the social context of infant oil massage using a theory of change approach. The process of development of the intervention has been covered into stages such as design, decide and implement. The design phase describes how the idea of nanosized liposomal encapsulated micronutrient fortified (LMF) body oil was conceptualized and how its feasibility was assessed through initial formative work in the community. The decide phase describes steps involved while scaling up technology from laboratory to community level. The implementation phase describes processes while implementing the intervention of LMF oil in a community-based randomized controlled study. Overall, the theory of change approach helps to outline the various intermediate steps and challenges while translating novel technologies for transdermal nutrient fortification to community level. In our experience, adaptation in the technology for large scale up, formative work and pilot testing of innovation at community level were important processes that helped in shaping the innovation. Meticulous mapping of these processes and experiences can be a useful guide for translating similar innovations.

## Introduction

Globally, almost 50% of under-five children suffer from hidden hunger due to deficiencies in essential nutrients, with majority of these children being from developing countries (1). In India, 58.5% of children aged 6–59 months are anemic (2) and 72% of this proportion is attributed to iron deficiency (3). Deficiencies of iron, folate and vitamin B12 especially during early childhood have been linked with poor cognitive development in children (2–4). Vitamin D deficiency is found in 40–99% of the Indian population of all ages and may have adverse health effects due to its important role in skeletal and extra-skeletal formation (5). Despite the well-recognized benefits of micronutrient supplementation interventions, the benefits of conventional oral supplements have been suboptimal due to poor adherence and concerns about safety and adverse effects (6). There is scope for development of innovative techniques for supplementation of these essential micronutrients.

This paper describes the journey of developing an innovative intervention for prevention of micronutrient deficiencies in children. The intervention involved use of a nanotechnology platform in the form of nanosized liposomes for fortification of body oil which is traditionally used for body massage in Indian infants. The intervention of nanosized liposomal encapsulated micronutrient fortified body oil (LMF body oil) was developed as part of a proof of concept randomized controlled study conducted among rural Indian infants to evaluate the effects of such an intervention on micronutrient deficiencies and neurodevelopmental outcomes.

In order to develop a complex intervention for healthcare delivery, it is important to understand the social context, acceptability and final sustainability which may have a significant effect on the outcomes (7, 8). Development of complex social interventions for mitigating health problems in a project mode or at health policy level essentially involves iterative intermediate steps for conceptualization, designing or monitoring and evaluation of the intervention (8). A Theory of Change (ToC) approach explains how different activities are understood to produce a series of results that contribute to achieving the final intended impacts (7). It is empirically tested by measuring indicators for every expected step along the hypothesized causal pathway to impact. The pathway is developed in collaboration with stakeholders and is modified in the process of development and evaluation through a process of reflection to explore the changes that happened during actual implementation (8).

Using the ToC approach, we describe various steps in the process of development and implementation of the intervention: (i) design (the construction of an intervention); (ii) decide (the decision making processes while developing the intervention); (iii) implement and monitor (covers all steps of practical implementation, tracking progress and allocation of supervision).

## Methods

The intervention of LMF body oil was developed and implemented for a proof of concept community-based clinical study in rural Indian children. This was double-blind placebo controlled randomized study in 444 infants to evaluate whether use of this intervention during 1st year of life can improve nutritional anemia and neuromotor development. The intervention of LMF oil was developed by researchers at Indian Institute of Technology Bombay (IITB), Mumbai, India and the study was implemented at in population of 22 villages of Vadu health and demographic surveillance (Vadu HDSS) (9) by researchers from Vadu Rural Health Program, KEM Hospital Research Centre (KEMHRC), Pune, India.

The causal pathway for ToC was developed through discussion with stakeholders involved in the development and implementation of the intervention. The initial pathway was presented in a workshop on “Effective Delivery of Integrated Interventions in Early Childhood” organized by Saving Brains Learning Platform, amongst other peer groups of various projects and was modified through feedback from peers and reviewers. The final pathway was developed after iterative inputs from the key stakeholders involved in the study i.e., investigators from partner institutes and implementers of the intervention. As this was a proof of concept project, the pathway focusses on the design, decide and implementation part of the process and does not largely cover monitoring and evaluation part of the ToC cycle. The pathway was developed through backward mapping of the activities that led to certain evidence-based decisions and changes in the work plan.

## Results

**Figure 1:**
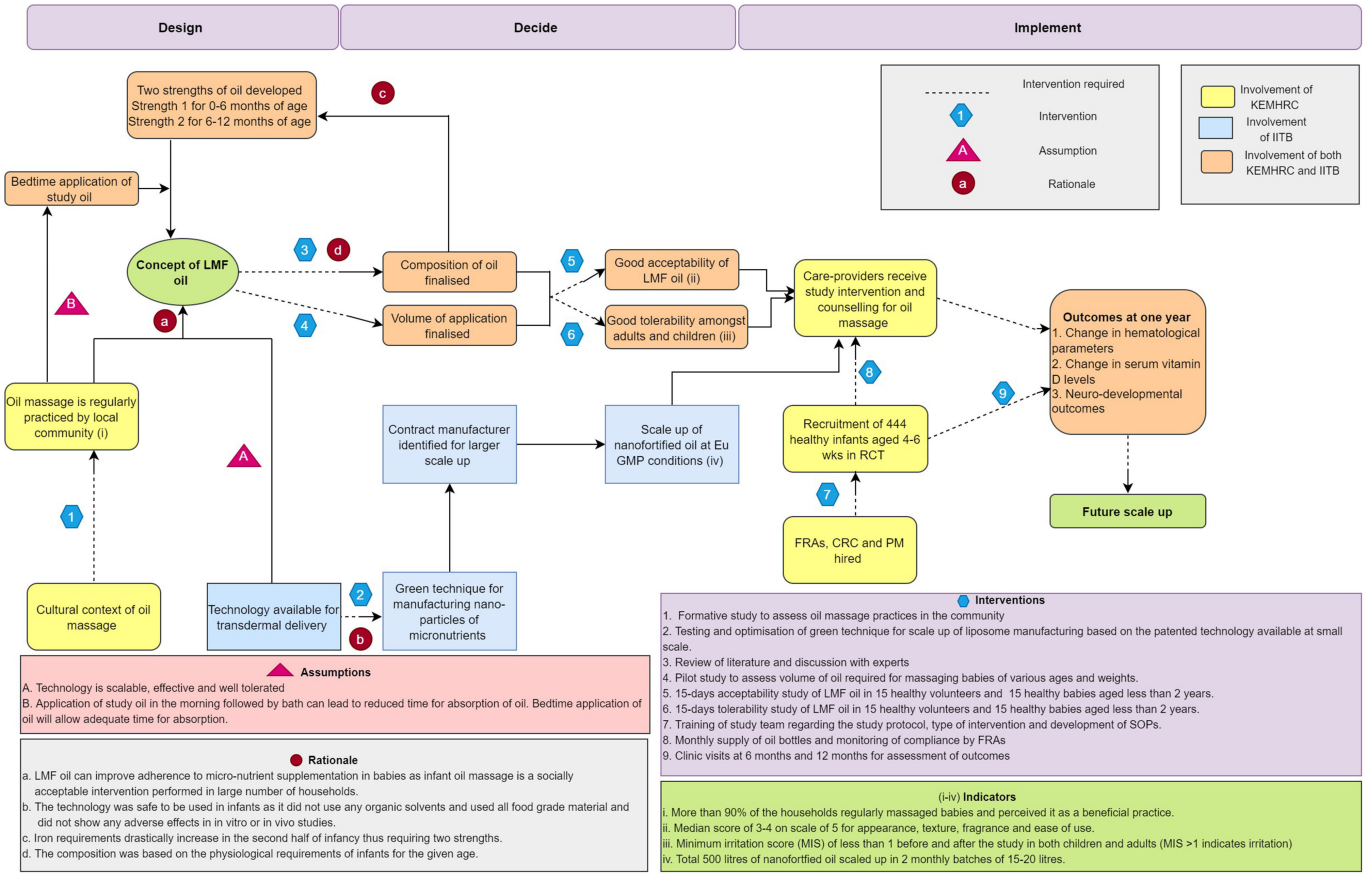
Causal pathway depicting theory of change approach for nanosized liposomal encapsulated micronutrient fortified body oil project. LMF body oil, Nanosized liposomal micronutrient fortified body oil; CRC, clinical research coordinator; PM, project manager; FRA, field research assistant; SOP, standard operating procedures; Eu GMP, European Good Manufacturing Practices; RCT, randomized controlled study; KEMHRC, King Edward Memorial Hospital Research Centre; IITB, Indian Institute of Technology Bombay.

Figure 1 displays the causal pathway developed for the project using the ToC approach. Decisions for finalizing processes during each stage of the project were taken through meetings with study staff and collaborators. The pathway also shows inputs from collaborating institutes at various phases of innovation. The results section describes how ToC applies to each phase of the innovation.

### Intervention Design

#### Cultural Context for Infant Oil Massage

Newborn oil massage is an ancient cultural practice in South Asian countries with some reported health benefits (10, 11). The available literature suggests that traditional oil massage is practiced among 80–90% of infants in South Asia, irrespective of the socioeconomic status or place of residence, and is initiated within a few days after birth. The most common perceived benefits of infant oil massage in the community include promotion of strength and health and maintenance of warmth (11, 12). Further, there is recent evidence about benefits of using emollients in newborns especially preterm for improving weight gain, reducing the risk of infection and associated mortality (13, 14).

#### Nanotechnology for Transdermal Delivery of Micronutrients

Nanoparticles are particles of 1–100 nm in size which exhibit unique properties due to the increase in the surface area to volume ratio of the nanoparticles (15). Liposomes are a type of nanoparticles that are spherical phospholipid based bilayers that self-assemble to entrap a hydrophilic medium in its core. They resemble the cell membranes in composition and can be used to co-entrap both hydrophilic and hydrophobic agents and have been extensively used in drug delivery. Suitably designed liposomes can potentially fluidize the stratum corneum by interacting with the ceramide rich lipid layer of the stratum corneum and penetrate through the intact skin and also along the hair follicles and sweat glands (16). The technology for transdermal delivery of nutrients using fluidizing phospholipid based liposomes has been developed by Banerjee et al. from Nanomedicine laboratory, Department of Biosciences and Bioengineering, IITB, Mumbai, India and has been patented (17). The technology has demonstrated safety and efficacy in *in vitro* and *in vivo* animal models for transdermal delivery of nutrients at supplemental doses (18). The technology has also demonstrated no irritation potential during irritation patch testing in healthy human volunteers (19).

#### Concept of Nanosized Liposomal Encapsulated Micronutrient Fortified Body Oil

LMF body oil is a body oil fortified with liposomes of nutrients (iron, folate, vitamin B12, and vitamin D) that can penetrate through the intact skin while massaging with the fortified body oil. This concept of LMF body oil emerged through initial meetings amongst scientists from KEMHRC and IITB. It was perceived that as the concept was based on the traditional practice of infant oil massage, it was likely to receive greater compliance and acceptability in the Indian community and can potentially overcome barriers associated with non-compliance to oral micronutrient supplementation (Figure 1, see Rationale-a). It was assumed at this stage that the technology would be feasible to scale up, tolerable and efficacious in transdermal delivery (Figure 1, see Assumption-A).

A proposal was developed through collaboration of the two research groups from two institutes (KEMHRC and IITB) for proof of concept evaluation of LMF body oil in a community-based project for improvement in neurodevelopment, prevention of iron deficiency anemia and vitamin D deficiency in rural Indian children. This was an interdisciplinary collaboration between technology scientists and medical as well as public health scientists. The proposal received funding from Saving Brains Platform of Grand Challenges Canada (20).

#### Formative Study to Assess the Practices of Infant Oil Massage in the Given Community

Before implementation of the study, a formative study was conducted in the study area for the community-based clinical trial (21). This was a questionnaire survey conducted amongst 201 households in the study area having infants below 2 years of age (Figure 1, see indicator (i)). The households were identified through line-listing of households using available data from Vadu HDSS. The objective of the study was to understand the local practices of infant massage and feasibility of implementing the study.

The caregivers in the household were asked by field research assistants about whether they practiced oil massage for their infants, age of onset for oil massage, till what age was the massage practiced, frequency of massage, choice of oil and perceived benefits of oil massage. The survey results supported the available evidence on oil massage as more than 90% of the caregivers practiced oil massage for their infants, believed that it was useful and also showed willingness to switch to a new oil formulation if that contained micronutrients. Majority of the households (about 65%) used marketed oils for massage. In all the households, infants were massaged in the morning before bathing. In 85% households, massage was initiated within the first 2 weeks after birth and in 94% households the massage was continued up to at least 9 months of age. This information demonstrated feasibility for implementation of study oil massage throughout the 1st year of life, especially in the second half of infancy when the prevalence of nutritional deficiencies rises.

### Decide

#### Finalizing Composition of LMF Body Oil

The composition of LMF body oil was finalized through review of literature and discussion with subject experts. The composition was devised taking into consideration the physiological requirements of the micronutrients with reference to the available national nutritional guidelines such that doses of none of the nutrients exceed 100% dietary requirement for the given age (21).

The physiological requirements of iron extensively rise after initial 6 months of life, whereas the iron requirements are minimal in the first 6 months due to redistribution of iron from fetal hemoglobin and supply of highly bioavailable iron through breastmilk (22). Physiological requirements for other nutrients i.e., folate, B12, and vitamin D remain similar during 1st year of life (21, 23, 24). With this, it was decided to formulate two compositions for oil (Figure 1 see Rationale-c), one for first 6 months and the other from 6–12 months with increased amount of encapsulated iron as compared to the initial strength (Supplementary Table 1).

Results from the formative study indicated that the local community was not in practice of using any particular natural oil, as majority of households used some form of marketed body oil. Based on review of literature regarding the choice of oil for infant massage, it was decided to use sunflower seed oil as this is a rich source of essential fatty acids and has demonstrated benefits in reducing morbidity and mortality in preterm newborns (25).

#### Revisiting the Volume of Oil and Time of Oil Massage

The regular practice of oil massage in the local community involved massaging infants in the morning, although some households practiced massaging at bedtime as well. The formative study demonstrated that the infants were bathed soon after this massage. It was felt that this practice may not provide adequate time for absorption of the liposomes due to wash off during bathing. Hence, we planned to implement the LMF body oil massage intervention at night before bedtime (Figure 1 see Assumption-B). This would provide night hours for absorption through skin and body massage at bedtime could potentially help the babies get sound sleep at night (26).

The LMF body oil was planned to be used throughout 1st year of life, during which the baby grows in size and body surface area changes which can lead to change in the volume of oil used for body massage. Also, there may be variability in the volume of oil used by each caregiver for a given size of a baby. It was important to finalize and fix the unit volume of oil to be used daily which would deliver the required dose of nutrients. A pilot study was carried out in 10 infants of various ages which demonstrated that sunflower seed oil in the volume of 2.5 mL was adequate to massage torso and extremities in first 6 months and was just adequate to massage extremities in the 6–12 month old babies (Figure 1 see Intervention-4). Thus, unit volume of 2.5 mL was decided for daily application in infants. There was also evidence from earlier literature that instillation of oil drops into nostrils may be associated with lipoid pneumonia (27). Hence, a decision was taken to only massage torso and extremities without applying oil to face and scalp.

#### Scaling Up Technology for Large Scale Manufacture of LMF Body Oil

The patented nanotechnology for preparation of micronutrient nanoparticles was available at laboratory scale and used organic solvents (methanol) for preparation of the liposomes. Additionally, the technique used (thin film hydration) was difficult to scale up for large scale manufacturing (28, 29). We therefore switched to a green technique of making liposomes using an organic solvent-free process using high speed homogenizers and the process was optimized to manufacture liposomes at large scale (Figure 1 see Rationale-b) (30). This technique was optimized and standardized inhouse. Various industry partners were approached for contract manufacturing and a EuGMP compliant service provider was identified for large scale production of the LMF body oil (Figure 1 see Indicator-iv). The technology was licensed to KEM Hospital Research Centre Vadu for non-commercial use and to Murli Krishna Pharmaceuticals Ltd. for manufacturing and commercialization.

The storage stability of the LMF body oil was also evaluated. Suitable packaging was used for providing monthly supply of LMF with instructions for storage away from light and heat. Calibrated cups were provided for measurement of accurate volumes of LMF. As the oil was to be used for a double blind randomized controlled study, test and placebo oils as well as low and high strengths of the oil were color-coded without disclosing the composition.

#### Tolerability of LMF Body Oil in Volunteers and Babies

We decided to evaluate the tolerability of LMF body oil in healthy adults and subsequently in healthy children before proceeding with the randomized controlled study in infants to rule out any irritation potential of the LMF body oils. The need for conducting these studies was perceived after discussion with subject experts as there is some earlier evidence on role of iron in oxidative stress in skin tissue (31) and further the approach of nanosized liposomes were an innovative platform for transdermal nutrient delivery (Figure 1 see Intervention-6). The studies were conducted after obtaining permission from institutional ethics committee and involved detailed written informed consent of study participants aged 18 yrs and above as well as from the parents of children who participated in the study. There was equal distribution of males and females while recruitment.

The first study was conducted in 26 healthy adult volunteers using single application of closed patch test (containing the product) under occlusion for 24 h. Subsequently, tolerability studies were conducted in 15 healthy adults and 15 healthy children aged 8–24 months where predefined quantity of highest strength of the LMF body oil was applied locally on the extremities for 15 days. In all the above studies, skin erythema and dryness were measured before and after the study using Draize score (32). The highest strength of the formulation did not show any irritation potential in any of the above studies (Supplementary Table 2).

#### Acceptability Studies

Before proceeding for the use of randomized controlled study, acceptability of using LMF body oil was assessed amongst the participants of the two tolerability studies. Acceptability of the oil was assessed for its appearance, texture, fragrance, ease of use and overall evaluation using visual analog scale of 0 to 5 where 0 indicates poor acceptability and 5 indicates best acceptability (Figure 1 see Intervention-5). The median score for all the categories were three or four with none of the observations were <2. This showed that the LMF body oil had good acceptability in view of the caregivers.

### Implement and Monitor

The community-based study consisted of administration of LMF body oil and a placebo oil to 222 infants respectively starting from age of 4–7 weeks till completion of 12 months. The implementation at the manufacturer level involved manufacturing of about 500 liters of oil including both test and placebo oil following the good manufacturing practices. At field level, a team of field research assistants, clinical coordinators, laboratory coordinator and project manager were hired and trained for the study protocol. Study documents including case record forms, participant information sheet, informed consent forms, logs for study activities and compliance cards were developed by the implementation team. The study team was trained for completing all the study activities as per the decided timeline.

During implementation of the project, the caregivers were trained about massage with LMF body oil at baseline visit in the study clinic. The field research assistants were trained to supply oil bottles on a monthly basis and monitor the intervention through fortnightly visits as well as through use of compliance cards. Additionally, during implementation, we found that checking the quantity of unused oil was a good measure of compliance. For non-compliant households, despite regular follow up by field research assistants, additional visits and counseling by a senior project staff were required and was found to facilitate compliance in majority of the households.

### Challenges Encountered

There were certain challenges encountered during the various phases of work. The laboratory technique available for preparation of nano-encapsulated liposomal micronutrients needed to be changed and a green technique needed to be developed and optimized for further scale up. Identification of the service provider for scaling up an innovative technology was a challenging task. Transdermal supplementation of nutrients is an innovative mode of delivery. Although technology has been tested in preclinical and clinical studies for efficacy and tolerability, exact proportion of absorption of encapsulated nutrients through pediatric skin is not known. Lastly, there were various challenges during implementation which have been depicted in Table 1. Community engagement of pregnant women for study participation, counseling of family members by study team for maintaining good compliance and use of compliance cards were measures taken to mitigate these challenges.

**Table 1:** Challenges encountered during project implementation.

**Recruitment**	**Compliance to intervention**	**Follow up study visits**
• Mothers visiting their maternal house during pregnancy • Social custom of not leaving the house till completion of post-partum period	• Sickness of the child • Resistance from one or more family member • Lack of interest • Lack of time	• Out migration • Fear of blood collection • Birth of next child

## Discussion

The intervention of a body oil that contains nanosized liposomes of micronutrients is a completely innovative intervention that was developed from laboratory-based technology to a proof-of-concept community level project. Transdermal iron replenishment using biophysical techniques such as microneedle patches and iontophoresis in *in vivo* animal models of anemia has been reported in the literature (33–35). However, no clinical studies have been reported in published literature. Scalability and costs involved due to additional infrastructure for external application of current scale limit its translation. Transdermal vitamin D supplementation using topical skin cream has been demonstrated in a pilot study in medical students (36). There is no published literature on transdermal supplementation of folate or vitamin B12 in clinical studies. Our earlier studies have reported the proof of concept preclinical studies for delivery of folate trans-dermally through cosmetics (18). Thus, this was one of the first attempts to develop and scale up a technology-based intervention for transdermal supplementation of multiple micronutrients through a body oil platform. Presently, the randomized controlled study has been completed with demonstrated improvement in micronutrient deficiency levels.

During development and implementation of LMF body oil, various aspects of the intervention needed to be addressed. These included feasibility of scaling up the intervention and implementing it, assessing acceptability by the local population and overall safety of the innovative intervention as well as developing measures to improve compliance to the intervention. This being a complex health intervention, lack of effect can be due to failure of implementation rather than genuine ineffectiveness (37). Hence, the process evaluation was necessary to understand the various links in the causal chain.

Our experience of using a causal pathway for ToC for development and implementation of LMF oil has several learnings (Table 2). The causal pathway shows that research ideas may be based on certain assumptions, but the assumptions need to be pilot tested while implementing. The outcomes which emerged during these intermediate steps have changed the directions of research and have modified the design of the innovation. The causal pathway also informed about importance of formative research work while developing and implementing a complex social innovation.

**Table 2:** Learnings while development of nanosized liposomal encapsulated micronutrient fortified body oil.

1	Collaborative discussions between interdisciplinary research teams
2	Modifications in the technology platform for scale up of technology innovation
3	Importance of formative work for testing feasibility of implementation
4	Importance of pilot testing while translating from laboratory based innovation to community level
5	Field-based adaptations to lay down the innovation on a traditional practice

While developing the innovation through the collaboration of scientists from technology and public health domains, the causal pathway helped to map steps where collaborative inputs from all stakeholders were useful in scaling up and evaluation (Figure 1). This also provides a useful example of how intermediate outcomes need to be assessed and how to overcome challenges that can be encountered during translation of technologies from laboratory scale to community level.

One of the limitations while developing this causal pathway was that it was developed retrospectively. Secondly, although the initial design and decide phase of the work was not dependent upon the external funding, it was carried out only after funding for the proof of concept study was available, thus leaving less time to carry out certain study related activities. Despite the limitations, the ToC approach for this project describes how a technology innovation can be based on a traditional practice and can be translated into a viable public health intervention.

In future, we plan to mechanistically evaluate the transdermal penetration of each ingredient of the oil through *ex vivo* studies and studies in human volunteers and also evaluate the efficacy of LMF oil in anemic children. We plan to scale up the manufacturing of LMF oil for larger public health use through transition to scale programs which will include monitoring and evaluation part of ToC cycle.

## Conclusion

Use of the causal pathway helped to clearly understand the intermediate steps taken and barriers faced during development of an innovation for transdermal delivery of nutrients, leveraging a traditional practice and its pilot implementation at community level. In our experience, adaptation in the technology for large scale up, formative work and pilot testing of innovation at community level were important processes that helped in shaping the innovation. Meticulous mapping of these processes and experiences can be a useful guide for translating similar innovations.

## Data Availability Statement

The raw data supporting the conclusions of this article will be made available by the authors, without undue reservation.

## Ethics Statement

The studies involving human participants were reviewed and approved by Institutional Ethics Committee, KEM Hospital Research Centre, Pune. Written informed consent to participate in this study was provided by the participants' legal guardian/next of kin.

## Author Contributions

AA, AB, SJ, and RB were involved in conceptualization of the research idea and writing of proposal. AA, AB, and RB were involved in designing of innovation. AA, MK, and RB were involved in technology scale up of the innovation. AA, HL, AB, and SJ were involved in implementation of the innovation at community level. AA attended the ToC workshop organized by Saving Brains Learning Platform and wrote the first draft of the manuscript. All authors reviewed and provided inputs on the causal pathway developed and read and agreed with the manuscript and conclusions.

## Conflict of Interest

The authors declare that the research was conducted in the absence of any commercial or financial relationships that could be construed as a potential conflict of interest.
