# Supplementary material for: Development and Implementation of Liposomal Encapsulated Micronutrient Fortified Body Oil Intervention for Infant Massage: An Innovative Concept to Prevent Micronutrient Deficiencies in Children
Source: Front Public Health. 2021 Jan 25;8:567689. doi: 10.3389/fpubh.2020.567689 (PMC7874153; doi:10.3389/fpubh.2020.567689)
Supplement: Supplementary file 1 [file Table_1.docx]

*Supplementary Table 1 : Composition of liposomal encaspulated micronutrient fortified body oil*

| **Composition per unit dose (2.5ml)** | **1-6 months** | **6-12 months** |
| --- | --- | --- |
| Elemental iron | 0.5 mg | 1.5 mg |
| Folate | 40 mcg | 40 mcg |
| Vitamin B12 | 0.3 mcg | 0.3 mcg |
| Vitamin D3 | 400 IU | 400 IU |

Base oil used : sunflower seed oil
