# Supplementary material for: Development and Implementation of Liposomal Encapsulated Micronutrient Fortified Body Oil Intervention for Infant Massage: An Innovative Concept to Prevent Micronutrient Deficiencies in Children
Source: Front Public Health. 2021 Jan 25;8:567689. doi: 10.3389/fpubh.2020.567689 (PMC7874153; doi:10.3389/fpubh.2020.567689)
Supplement: Supplementary file 2 [file Table_2.docx]

*Supplementary Table 2: Tolerability and acceptability of nanosized lipo-encapsulated micronutrient fortified body oil in adults and children*

| **Type of study** | **Baseline MIS** | **Post-completion MIS** | **Irritancy assessment** |
| --- | --- | --- | --- |
| 24-hrs irritation patch test amongst adults (n=26) | 1.53 | 0 | Non-irritant |
| 15-days tolerability in adults (n=15) | 0 | 0.033 | Non-irritant |
| 15-days tolerability in children (n=15) | 0.0769 | 0 | Non-irritant |

MIS – Mean irritation score assessed using Draize’s scale which mainly assesses erythema and dryness at the site of local application, Interpretation - <2- non-irritant, 2-4 – Mild irritant, >4 – Irritant.
